# Impaired immunological synapse in sperm associated antigen 6 (SPAG6) deficient mice

**DOI:** 10.1038/srep25840

**Published:** 2016-05-12

**Authors:** Lauren Folgosa Cooley, Mohey Eldin El Shikh, Wei Li, Rebecca C. Keim, Zhengang Zhang, Jerome F. Strauss, Zhibing Zhang, Daniel H. Conrad

**Affiliations:** 1 Center for Clinical and Translational Research (CCTR), Virginia Commonwealth University, Richmond, VA 23298, USA; 2 Department of Microbiology and Immunology, Virginia Commonwealth University, Richmond, VA 23298, USA; 3 Experimental Medicine and Rheumatology, William Harvey Research Institute, Queen Mary University of London, Charterhouse Square, London, EC1M 6BQ, UK; 4 Department of Obstetrics and Gynecology, Virginia Commonwealth University, Richmond, VA 23298, USA; 5 Department of Infectious Diseases, Tongji Medical College, Huazhong University of Science and Technology, Wuhan, Hubei, China

## Abstract

Sperm associated antigen 6 (SPAG6), a component of the central apparatus of the “9 + 2” axoneme, plays a central role in ciliary and flagellar motility; but, its contribution to adaptive immunity and immune system development is completely unknown. While immune cells lack a cilium, the immunological synapse is a surrogate cilium as it utilizes the same machinery as ciliogenesis including the nucleation of microtubules at the centrosome. This prompted our hypothesis that SPAG6 critically regulates the formation and function of immunological synapses. Using bone marrow reconstitution studies of adult WT mice, we demonstrate that SPAG6 is expressed in primary and secondary lymphoid tissues, is associated with the centrosome in lymphocytes, and its deficiency results in synapse disruption due to loss of centrosome polarization and actin clearance at the synaptic cleft. Improper synapse formation in *Spag6*KO mice was associated with defective CTL functions and impaired humoral immunity as indicated by reduced germinal centers reactions, follicular CD4 T cells, and production of class-switched antibody, together with expansion of B1 B cells. This novel report demonstrates the requirement of SPAG6 for optimal synapse formation and function, its direct role in immune cell function, and provides a novel mechanism for infertility disorders related to SPAG6.

Mammalian sperm associated antigen 6 (SPAG6), or its orthologue, PF16 protein, from *Chlamydomonas reinhardtii*, is classically known as a component of the central apparatus of the “9 + 2” axoneme, which consists of a central pair of microtubules (C1 and C2) surrounded by nine outer microtubule doublets with associated dynein arms^1^,^2^,^3^. Mutagenesis of *Chlamydomonas* PF16 results in flagellar paralysis and disturbance of C1 central microtubule stability revealing its central role in flagellar stability and motility^3^. In mammals, SPAG6 is widely expressed, mainly in tissues with cilia-bearing cells including lung, nervous system, inner ear, and particularly, testicular germ cells where SPAG6 resides in the sperm flagella^1^,^4^. Many of the noted abnormalities associated with SPAG6 deficiency are related to dysfunctional ciliary or flagellar appendages in ciliated cells and tissues. In humans, SPAG6 in the sperm tail is targeted by a class of anti-sperm autoantibodies associated with immune-mediated infertility in males^4^. Global SPAG6-deficient mice (*Spag6*KO) exhibit significant defects including a high percentage of neonatal deaths, hydrocephalus, and infertility in surviving male mice^2^. They also experience a significant reduction in axoneme numbers and the two central microtubules exhibit a random orientation compared to WT mice^5^. Furthermore, *Spag6*KO tracheal epithelial cells lose their polarized morphology and polarized distribution of mucin; and these phenotypes appear to be related to a disrupted microtubule system^5^. In addition, *Spag6*KO mice develop otitis media more readily than WT given abnormalities in the ciliated columnar epithelium in the middle ear and Eustachian tube^6^. While these examples demonstrate the importance of SPAG6 in ciliated tissues, the role of SPAG6 in cells lacking classic cilia and/or flagella, such as lymphocytes or other immune cells, remains unreported. SPAG6 is a tubulin-binding protein, and in addition to its role in classic ciliogenesis, we hypothesize that it regulates several other microtubule/cytoskeletal functions required for the proper function of modified cilia such as immunological synapses. In fact, SPAG6 may be involved in a broad range of cellular functions including cell division, proliferation, migration, adhesion, and secretion. Support for this concept comes from our recent demonstration of abnormal fibroblast motility in SPAG6 deficient MEFs^7^. Another protein critical for ciliogenesis, IFT20, has been shown to be important for microtubule organization and T cell receptor (TCR) recycling at the immunological synapse^8^,^9^. Therefore, given the contribution of SPAG6 protein to microtubule organization, and the critical role of microtubule organization in cognate immune cell interaction, we sought to analyze the role of SPAG6 in the formation of the immunological synapse as well as the impact of SPAG6 deficiency on the immune response.

The microtubule organizing center (MTOC) is a subcellular apparatus that functions to form and organize microtubules, which in eukaryotic somatic cells is the centrosome, composed of two orthogonally arranged centrioles (mother centriole and daughter centriole)^10^,^11^. During an immune response, upon cognate recognition between an antigen presenting cell and effector cell, reorientation of the centrosome, actin, the Golgi, and secretory vesicles occurs in the effector cell at the immunological synapse, allowing receptor/ligand interactions and targeted release of cytokines^12^. Furthermore, during targeted killing by an effector cell, the same reorientation occurs with docking of the centrosome at the synapse membrane of the effector cell, effectively creating the synaptic cleft by which cytolytic enzymes are released for targeted destruction^13^,^14^. While SPAG6 is known to be a microtubule-binding protein^1^ and co-localizes with polymerized microtubules^14^, we demonstrate, herein, the expression of SPAG6 protein in primary and secondary lymphoid tissues, its association with the centrosome, and the lack of centrosome polarization and actin clearance at the synaptic cleft of SPAG6 deficient mice. In addition, we show that these synaptic malformations are associated with reduced CTL cytotoxicity, diminished follicular CD4 T cell retention in germinal centers, defective germinal center reactions, low production of class-switched antibodies, and expansion of the B1 B cell subset.

## Results and Discussion

### SPAG6 is expressed in primary and secondary lymphoid tissues and is associated with the centrosome in transfected and non-transfected HEK cells as well as WT lymphoid cells

**Figure 1:**
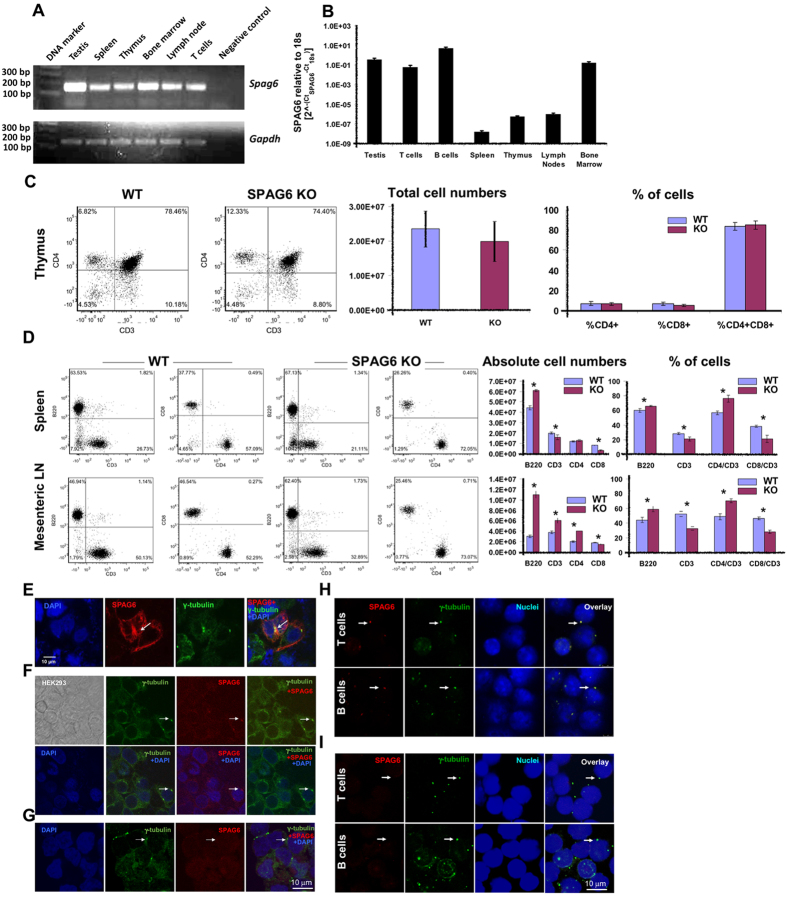
SPAG6 expression in lymphoid cells and tissues and presence of SPAG6 protein in the centrosome. (**A**) SPAG6 expression in adult WT mice compared to testis (positive control) and negative control (water). SPAG6 is expressed in primary (thymus, bone marrow) and secondary (spleen, lymph node) lymphoid tissues and T cells. (**B**) Quantitative *Spag6* expression in different lymphoid tissues assessed by qRT-PCR and normalized relative to the 18s housekeeping gene. (**C**) Dot plots and histograms showing the absolute numbers and percentages of T cell subsets in the WT and *Spag6*KO thymic tissues. (**D**) Dot plots and histograms showing the absolute numbers and percentages of B cells (B220) and T cell subsets in the WT and *Spag6*KO spleens and lymph nodes [*p < 0.05]. (**E**) HEK293 cells were transfected with *Spag6*/pcDNA3 plasmid, and the cells were stained for DAPI (blue), SPAG6 (red) and γ-tubulin (green), a component of centrosome. Co-localization of SPAG6 and γ-tubulin is indicated by white arrows. (**F**) Non-transfected HEK293 cells were stained for DAPI (blue), SPAG6 (red) and γ-tubulin (green). The cells were also imaged using differential interface contrast (DIC) mode (upper left image). Co-localization of endogenous SPAG6 and γ-tubulin is indicated by white arrows. (**G**) Non-transfected HEK293 cells labeled with mouse anti γ-tubulin only (rabbit anti Spag6 primary Ab omitted) followed by staining with DAPI (blue), anti rabbit IgG (red) and anti mouse IgG (green). No co-localization of endogenous SPAG6 and γ-tubulin can be seen at the white arrows [negative control]. (**H**) Purified WT T and B cells were labeled with anti SPAG6 (red) and anti γ-tubulin (green). Co-localization of SPAG6 and γ-tubulin is indicated by white arrows. (**I**) Purified WT T and B cells were labeled with mouse anti γ-tubulin only (rabbit anti SPAG6 primary Ab omitted) followed by staining with DAPI (blue), anti-rabbit IgG (red) and anti-mouse IgG (green). No co-localization of SPAG6 and γ-tubulin can be seen at the white arrows [negative control].

The expression and role of SPAG6 in immune system development and adaptive functions is unknown. While the murine immune system fails to mature until about six weeks of age, the majority of *Spag6*KO mice survive less than three weeks due to numerous developmental defects. Therefore, we first confirmed expression of SPAG6 in various immune cells and tissues of adult WT mice including spleen, thymus, bone marrow, lymph node and naïve B and T cells by qPCR (Fig. 1A,B). We then irradiated and reconstituted adult WT mice with WT or *Spag6*KO bone marrow from 7–14 day old mice in order to study immune system development and function following reconstitution.

Analysis of the absolute numbers and percentages of lymphocytes in different lymphoid tissues indicated that, despite insignificant difference between WT and *Spag6*KO in T cell distribution in the thymus (10–14 day old mice) (Fig. 1C), lymphocytes in peripheral lymphoid tissues (MLN and spleens) of adult reconstituted animals were different showing decreased CD8^+^ T cells and increased numbers of B cells (Fig. 1D). Defective lymphocyte homing to peripheral lymphoid tissues can be attributed to cytoskeleton/microtubule instability in Spag6KO mice. In fact, disordered lymphocyte migration has been described in several mouse models where cytoskeletal/motility-associated proteins have been knocked out^15^. Not only that, but conserved molecular pathways critically define and maintain a nucleus–centrosome axis parallel to the front-to-rear polarity axis of migrating cells^16^. Consequently, defective centrosomal structure and location due to SPAG6 deficiency may also contribute to inefficient cell migration and tissue homing.

SPAG6 is known to be critical for ciliogenesis and microtubule-based motility^5^; and despite the fact that lymphocytes do not have cilia, their engagement with antigen presenting cells and target cells involves extensive remodeling of cytoskeletal and motor proteins. De la Roche *et al.* proposed that the T cell immunological synapse at the interface between T cells and antigen presenting/target cells is a “surrogate cilium” because it utilizes the same machinery as ciliogenesis including the nucleation of microtubules at the MTOC or centrosome^12^. De la Roche *et al.* also described how Hedgehog signaling, originally known for its role in primary cilia formation, is also critical for CTL function and immunological synapse formation^12^. Consequently, we sought to determine if SPAG6 is present in the MTOC or centrosome and, if so, could SPAG6 be required for proper immunological synapse formation and function. We previously reported that SPAG6 decorated and appeared to organize the microtubules in transfected CHO cells^14^, however, whether SPAG6 protein is a structural component of the MTOC or centrosome is not known. To explore the SPAG6-centrosome association, HEK293 cells were transfected with SPAG6/pcDNA3 plasmid and then the cells were double labeled with a polyclonal antibody against SPAG6 and a monoclonal antibody against γ-tubulin, a centrosome component. As shown in Fig. 1E,F, SPAG6 co-localized with γ-tubulin indicating that SPAG6 protein is structurally associated with the MTOC/centrosome apparatus. Furthermore, we sought to investigate the association of SPAG6 and the centrosome marker γ-tubulin in lymphocytes. Purified B and T cells were labeled with anti-SPAG6 and γ-tubulin and as shown in Fig. 1H, the two proteins were associated in WT lymphocytes. Negative controls where the anti-SPAG6 Ab was omitted showed no SPAG6 labeling in HEK293 (Fig. 1G) or lymphocytes (Fig. 1I). Compared to B and T cells, the residual background in HEK cells at the same imaging parameters seems to be higher due to autofluorescence. Autofluorescence is directly proportional to energetic metabolism and the proliferative activity of the cell^17^,^18^,^19^. In contrast to B and T cells, HEK is highly proliferative and kept in cultures longer which can contribute to the observed higher background. Streptavidin-biotin amplification was used for the detection of endogenous SPAG6 in HEK cells. The co-localization of SPAG6 and γ-tubulin was comparable to the non-amplified conditions and images have been included in supplementary Fig. 2.

### SPAG6 is required for centrosome polarization and actin clearance at the immunological synapse

**Figure 2:**
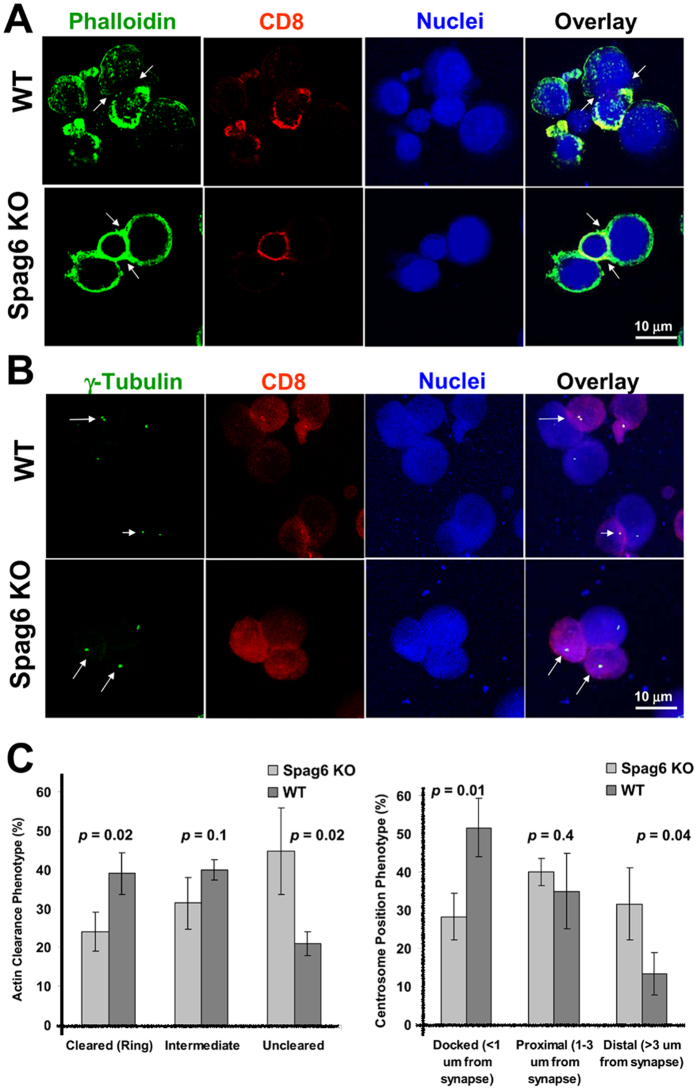
Defective actin clearance and centrosome polarization at the immunological synapse in *Spag6KO* mice. (**A**,**B**) The immunological synapse between WT or *spag6*KO CD8^+^ CTLs (red) and P815 target cells was stained for (**A**) F-actin (green, white arrows, upper panel) or (**B**) γ-tubulin (green dots, white arrows, lower panel). (**C**) Quantification of actin clearance (left) and centrosome (γ-tubulin) polarization and docking distance from the synapse in WT and *Spag6*KO CD8^+^ cells shown. P values are shown.

Given that SPAG6 is structurally associated with the centrosome (Figs 1C–E and 1C), and the centrosome is crucially involved in synapse organization, we predicted that SPAG6 plays a critical role in immunological synapse formation. Two hallmarks of adequate synapse formation are centrosome polarization to the synapse and actin clearance from the synapse^12^. At the central supra-molecular activation cluster of the immunological synapse, the centrosome moves to and contacts with the plasma membrane, whereas actin is cleared away from the synapse. It has been proposed that centrosome polarization might be driven by the reorganization of the actin cytoskeleton, clearing the plus ends of microtubules from the area of contact and pulling the centrosome towards the plasma membrane^20^. To analyze the role of SPAG6 in synapse formation, allogeneic CTLs were cultured briefly with P815 target cells, then labeled for F-actin and γ-tubulin to visualize the synaptic cleft at the interface between CTLs and their targets. Figure 2A,C demonstrates that SPAG6 is required for actin clearance at the synapse as lack of SPAG6 in *Spag6*KO CD8 T cells was associated with inefficient actin clearance and persistence of actin staining at the synapse. As seen in Fig. 2A and quantified in Fig. 2C, no polymerized actin (F-actin labeled with phalloidin) can be seen in the central region of the synapse in WT CD8 T cells only where actin filaments are actively cleared from the central regions of the synapse by de-polymerization^21^. Movies constructed from z stacks passing through the whole depth of the synapses confirmed lack of actin clearance in *Spag6*KO mice and efficient regression in the WT mice (Movie S1– WT and S2– *Spag6*KO). As seen in a series of still images taken from the supplementary movies [supplementary Fig. 3], the amount of actin displayed in still images varies at different focal planes. This explains the less displayed overall actin in the WT in Fig. 2A as the plane of maximum actin clearance at the immunological synapse co-existed with reduced actin staining.

Actin reorganization is thought to play a role in centrosome polarization^20^, consequently, lack of SPAG6 also resulted in improper polarization of the centrosome and lack of positioning at the synapse (Fig. 2B,C). Specifically, in WT CTL cells, centrosome docking at the synapse occurred in 50% of CTL/target cell synapses with only 10% positioned distal to the synapse; whereas, *Spag6*KO CTLs exhibited centrosome docking in only 30% of synapses (Fig. 2C).

### Reduced T cell cytotoxicity in *Spag6*KO

Thus far, we have determined that SPAG6 is expressed in primary and secondary lymphoid tissues (Fig. 1A,B), associated with the centrosome (Fig. 1E–I), and its deficiency is associated with defective centrosome polarization and actin clearance (Fig. 2, Movies S1 and S2) suggesting that these structural abnormalities will obstruct proper functional communication at the synapse site, which can be assessed by analyzing cytotoxic T cell (CTL) killing of target cells and T cell-dependent class-switched antibody production in WT and *Spag6*KO mice.

**Figure 3:**
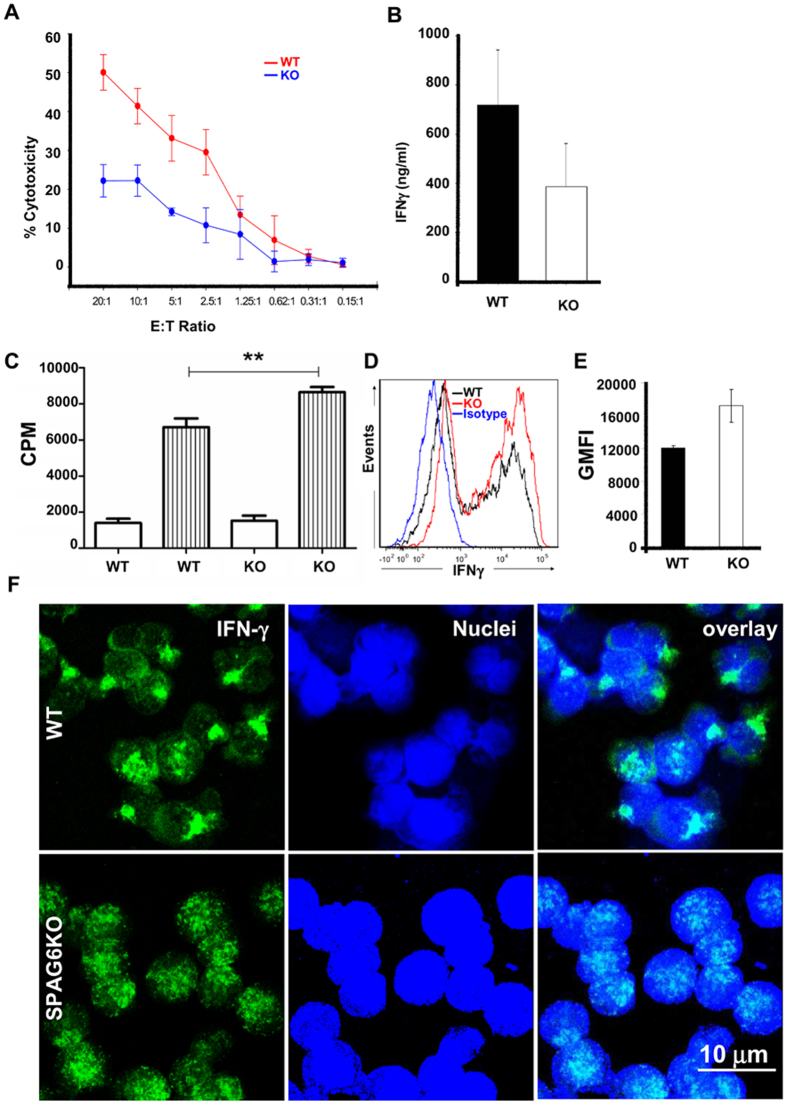
Reduced CTL function in *Spag6*KO mice. (**A**) Allogeneic cytotoxicity assay with decreasing ratios of WT (red) or *Spag6*KO (blue) CD8^+^ T cells to P815 target cells; (**B**) Soluble IFNγ production by activated WT or *Spag6*KO CD8^+^ T cells (**C**) WT or *Spag6*KO (KO) CD8+ T cell proliferation in media alone (white) or with CD3/CD28 stimulation (stripes). (**D**) Histogram showing intracellular IFNγ labeling in WT and *Spag6*KO CD8 T cells compared to isotype control. (**E**) The geometric mean of intracellular IFNγ fluorescence intensity (GMFI) is significantly higher in the *Spag6*KO CD8s compared to the WT (**F**) Intracellular IFNγ distribution in activated WT (upper panel) or *Spag6*KO (lower panel) CD8^+^ T cells. N = 3 – 7 per group; **p < 0.005.

CTLs destroy target cells following T cell receptor activation through release of secretory lysosomes at the immunological synapse. T cell receptor activation triggers migration of the MTOC to the contact site, causing the generated microtubule network to polarize towards the target^13^. Secretory lysosomes then travel in a minus end direction toward the MTOC followed by transient fusion of the centrosome with the plasma membrane to mediate secretion into the synapse^20^. Given the presence of SPAG6 in the centrosome, we reasoned that in the absence of SPAG6, reduced CTL-induced cytotoxicity would occur. We performed an allogeneic cytotoxic T cell assay and demonstrated that *Spag6*KO T cells had significantly reduced capacity to induce P815 cell death compared to WT CD8^+^ T cells (Fig. 3A). Using a paired Mann-Whitney test without assuming the normal distribution of the values, the *p* value was 0.016. using the mixed effects model^22^, WT mice showed 15 times higher cytotoxicity compared to *Spag6*KO mice. Furthermore, stimulated *Spag6*KO CD8^+^ T cells had reduced secretion of soluble IFNγ (Fig. 3B). These findings are not due to reduced T cell proliferation (Fig. 3C) or production of IFNγ by the *Spag6*KO CD8 cells (Fig. 3D,E). In fact, *Spag6*KO CD8 T cells cultured with anti-CD3/CD28 exhibit enhanced proliferation (Fig. 3C) and higher intracellular IFNγ (Fig. 3D,E) compared to WT CD8 T cells. It has been previously shown that IFNγ has a positive feedback effect on its own synthesis^23^ and release^24^. We reason that this positive feedback loop in WT cells together with defective secretion of IFNγ in *Spag6*KO cells resulted in higher retention and intracellular labeling of IFNγ in the KO cells. Moreover, we noticed that the reduction in IFNγ release from activated *Spag6*KO CD8^+^ T cells was associated with homogenous non-polarized distribution compared to WT (Fig. 3F). These findings strongly support the concept that reduced IFNγ release from activated *Spag6*KO CD8 T cells is due to defective secretion not production. SPAG6 is a tubulin-binding protein, and in addition to its role in classic ciliogenesis and the integrity of the immunological synapse, we reason that it can regulate several other microtubule/cytoskeletal functions including intracellular polarization and release of cytokines and lytic granules. Intracellular vesicle transport mechanisms are intimately linked to the microtubule cytoskeleton, and polarized vesicle traffic to the immunological synapse occurs via the polarization of the microtubule cytoskeleton^25^. In addition, the Golgi apparatus is associated with the MTOC and reorients with it following microtubule reorganization. Golgi polarization to the immunological synapse is critical to the polarized secretion of cytokines including IFNγ^20^. Only in the WT group was IFNγ polarized in the CD8^+^ T cells. The non-polarized distribution and lower release of IFNγ by *Spag6*KO CD8^+^ T cells strongly suggest a critical role of SPAG6 in cytokine polarization and delivery and we look forward to explore further functional consequences of SPAG6 deficiency in these areas.

### WT mice reconstituted with SPAG6 deficient bone marrow exhibit impaired GC formation, diminished follicular CD4 T cells, reduced class-switched antibody production, and expansion of B1 B cells

**Figure 4:**
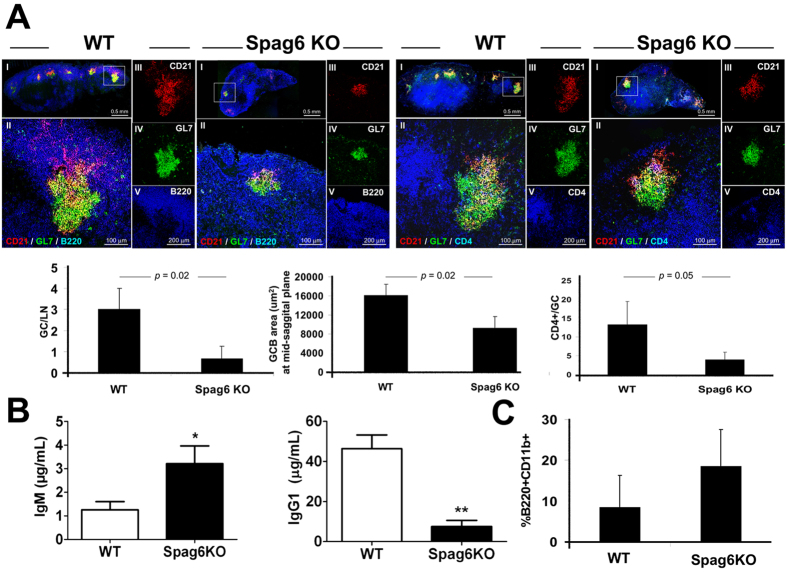
Defective humoral immune response in *Spag6*KO mice. (**A**) Reduced GC formation and GC CD4^+^ T cells in draining LN (day 14) of WT and *Spag6*KO reconstituted mice. (I) Low magnification image showing the whole LN at a mid saggital section. The GC in white box is shown at higher magnification in (II) and Separate channel recordings (III, IV, V) are provided to the right of the overlay of the presented GC. The left panel shows FDCs (CD21^+^, red), GCB cells (GL7^+^, green), and B220/CD45R (pan B cell marker, blue) in the lymph node cortex. The right panel shows FDCs (CD21^+^, red), GCB cells (GL7^+^, green), and CD4^+^ T cells including GC CD4^+^ T cells (blue). Morphometric analysis of GCs illustrated in histograms shows reduced number and size of GCs and CD4^+^ T cells per GC in *Spag6*KO. (**B**) Day 14 NP-KLH specific (**B**, left) IgM and (**B**, right) IgG1 levels in sera. (**C**) Percentage of peritoneal B1 B cells (B220^+^ CD11b^+^) collected at day 14 post-immunization. N = 4 − 6 per group, 3 independent experiments. *p < 0.05, **p < 0.005.

Establishment of a proper immunological synapse is required for optimal antibody production as well. A series of synapses is generated to mount an effective humoral immune response. First, a synapse between cognate antigen-specific B and T cells occurs at the B/T border to trigger downstream GC formation. Furthermore, synapse formation and signaling between B cells and Tfh cells is required for class switch recombination and affinity maturation^26^. Moreover, BCR engagement with FDC-retained antigens is critical for GC persistence and selection of high affinity B cell clones^27^. We, therefore, hypothesized that *Spag6*KO mice, would exhibit impaired GC formation and reduced class-switched antibody production post immunization with T cell-dependent antigens. Following immunization with the T cell-dependent NP-KLH antigen, the germinal center reactions and CD4^+^ T cells in the draining LNs were assessed in WT or *Spag6*KO reconstituted mice. Following 14 days of immunization, the number and size of GCs in the follicle were significantly reduced in *Spag6*KO (Fig. 4A). Furthermore, the number of GC CD4^+^ T cells were significantly diminished, suggesting reduced migration of Tfh cells to the GC perhaps due to lack of initial signaling/interaction with the B cell, or due to impaired retention in the GC due to lack of effective GCB cell/T cell synapse (Fig. 4A). In fact, stable GC-B cell/T cell interaction is critical for selection of antigen-specific B cells, B cell activation and plasma cell differentiation, and this interaction is associated with reduction in both GCB and Tfh cell velocities, along with an increase in the duration and size of the T-B contacts^21^,^28^,^29^,^30^.

The reduction in GC formation and CD4^+^ T cells in *Spag6*KO mice corresponded with significantly reduced NP-specific IgG1 production on day 14 (Fig. 4B). IgG1 dominates IL-4-dependent humoral Th2 responses characteristic of immune responses to immunization with alum-precipitated proteins. Having checked the impact of SPAG6 deficiency on IFNγ and CD8 cytotoxicity, two major arms of Th1-dominant inflammatory cell mediated responses, we assessed IgG1 levels to investigate the impact of SPAG6 deficiency on Th2 mediated humoral immunity. While NP-specific IgM and IgG1 levels were comparable at day 7 post immunization (data not shown), NP-specific IgM is increased in *Spag6*KO serum at day 14 (Fig. 4B, left). This result could be attributed to increased extrafollicular antibody production in *Spag6*KO B2 cells or perhaps due to the increase in peritoneal B1 B cells (Fig. 4C). B1 B cells are innate, self-renewing, long lived B cells, which typically reside in the peritoneal and pleural cavities as well as spleen^31^. They fail to enter germinal centers, are thought to make natural antibodies, and do not require cognate T cell interaction for IgM production^31^. In immunized mice the percentage of peritoneal B1 B cells (B220^+^ CD11b^+^) was 8.4 ± 7.8 and 18.6 ± 8.9 (Fig. 4C) in WT and *Spag6*KO mice, respectively, with a two-tailed p-value of 0.0775. Despite the lack of statistical significance, there is a trend in increased B1 B cells in *Spag6*KO mice, which might be attributable, as suggested, to proliferation of surviving peritoneal SPAG6-sufficient recipient B1 B cells, which are less sensitive to irradiation, or due to enhanced formation of B1 B cell precursors induced by SPAG6 deficiency. In the latter case, expression of SPAG6 would decrease the number of B1 B cells, providing an interesting target for limiting autoimmune reactions, as increased B1 B cells are related to predisposition to autoimmunity^32^,^33^.

In humans, SPAG6 is a ciliary protein critically involved in sperm motility, and is targeted by autoantibodies in immune-mediated male infertility^4^. Herein, our novel description of SPAG6’s role in the immune system reveals the potential comorbidity of immune deficiency and immune infertility. Impaired immunological synapse formation and function in patients with SPAG6-related immune infertility may precipitate chronic infections and inflammations of the male and female reproductive tract. Such chronic infections may further aggravate infertility by obstructing the reproductive tracts, decreasing immuno-modulatory factors that normally prevent sperm autoimmunization, or induce other anti-sperm autoantibodies due to cross-reactivity between microbial and sperm antigens^34^. Furthermore, empirical immuno-suppression by steroids has been used to treat patients with anti-sperm antibodies. Although steroids suppress antibody levels and may improve fertility in some male patients, most of the reported data suggests that steroid therapy may be ineffective for immune infertility^35^. Given that patients with SPAG6-related infertility may already be at risk due to immunodeficiency in view of our evidences of the importance of SPAG6 in immunological synapse function, prescribers should be aware of the potential additive immunosuppressive effect of corticosteroid therapy in this group of patients.

SPAG6 and its orthologue PF16 have long been considered ciliary/flagellar motility regulators. For example, it was shown that insertional mutagenesis of PF16 in *Chlamydomonas reinhardtii* causes flagellar paralysis^3^,^36^,^37^ ; and that inhibition of PF16 expression by RNA interference in *Trypanosoma brucei* results in flagellar detachment, loss of propulsive waves and motility^38^. In addition, sperms in SPAG6-deficient male mice have marked motility defects associated with disorganization of flagellar structures including loss of the central pair of microtubules^2^. Despite these clear ciliary/flagellar motility disorders, SPAG6-deficiency is also associated with phenotypes, such as growth-retardation and hearing loss, which cannot be entirely explained by ciliary/flagellar dysfunction. As a result, other SPAG6 functions, beyond motility regulation, were sought for, and data supporting such functions started to emerge^39^. For example, a role for SPAG6 in primary and motile cilia genesis and polarity has been recently suggested^5^, and we have recently shown that SPAG6 controls cytoskeleton/microtubule stability in mouse embryonic fibroblasts through regulation of tubulin acetylation^7^.

Compared to non-motile primary cilia, several integrated functions are performed at the immunological synapse. Immunological synapses are generated at the interface of different immune cells where vesicle transport and directed release of cytokines (e.g. IFNγ) and chemokines are essential for proper synapse functions. Lipid rafts, receptor mobility, microclustering and intracellular signalling are essential components of immunological synapses and required for immune cell activation and proper mounting of an immune response. In view of our recent studies, we believe that the primary function of SPAG6 is to modulate cytoskeleton/microtubule stability through regulation of structural and transport proteins, and consequently the impact of SPAG6 deficiency will be more evident in immunological synapses than non-motile cilia. Immunological synapses are orchestrated by and highly dependent on several arrays of cytoskeletal and transport proteins, and defective function of one or more of these proteins due to SPAG6 deficiency will impair synapse coordination and can be perpetuated thorough the system. IFNγ has a positive feedback effect on its own synthesis, and defective IFNγ release will impair its autocrine and paracrine activity. In addition, an effective immune response entails generation of a series of synapse formation in relatively short times, where failure of one will create a domino effect resulting in defective immune response.

In conclusion, while classically studied in ciliary and flagellar motility, SPAG6 is expressed in primary and secondary lymphoid tissues and its role in immune system development and function stems from its critical role in proper immunological synapse formation. We provide novel and substantial evidence that SPAG6 is associates with the centrosome in lymphocytes and is required for optimal centrosome polarization and actin clearance during synapse formation. SPAG6 deficiency is associated with inadequate synapse formation manifested by significant functional consequences including reduced CD8 cytotoxicity, CD8 T cell IFNγ secretion, GC formation, GC CD4^+^ T cell retention, and class-switched antibody production.

Overall, SPAG6, a protein classically studied in ciliary motility, is shown for the first time to play a critical role in immunological synapse formation and function. SPAG6 deficiency results in impaired synapse formation due to defective actin clearance and centrosome docking. Lack of effective synapse formation leads to synaptic dysfunction with detrimental consequences including reduced T cell cytotoxcity and class-switched antibody production. Therefore, the immune status of patients with SPAG6-related infertility should be evaluated, and the possible immunodeficiency needs to be considered in the management plans of these patients.

## Methods

### Mice

All animal care and experimental protocols were approved by VCU IACUC and in accordance with NIH guidelines. *Spag6*KO mice were generated as previously described^1^ and compared to WT littermate controls. C57/SV129 WT male mice age 6–8 weeks were used for reconstitution.

### Bone Marrow Reconstitution and NP-specific ELISA

C57/SV129 WT were irradiated and reconstituted i.v with 5 million bone marrow cells from WT or *Spag6*KO as previously described^40^,^41^. After 6 weeks, mice were footpad and i.p immunized with 10 μg 4-hydroxy-3-nitrophenylacetyl coupled to keyhole limpet hemocyanin at a ratio of 27:1 (Bioresearch Technologies) in 4 mg alum. Mice were bled at day 7 and organs harvested at day 14. NP-specific IgM and IgG1 was determined by ELISA using NP-14–BSA (15 μg/ml; Biosearch Technologies) as previously described^42^.

### Reagents, T cell culture, IFNγ ELISA

Total splenocytes were incubated with CD8-PE (Biolegend) and CD8^+^ T cells isolated by PE-positive selection using EasySep kit (Stemcell Technologies). T cells were grown in complete RPMI^34^ with 100 Units/mL IL-2 (Peprotech) on overnight anti-CD3ε (1 μg/mL, Biolegend) treated plates with anti-CD28 (2 μg/mL, Biolegend) and supernatants collected for IFNγ ELISA (eBioscience) after 72 h of growth. Proliferation was assessed after 72 h as previously described^41^. These data were obtained from 4–5 ^Spag6^KO or WT mice, and the experiments were repeated at least twice. Intracellular IFNγ was stained using biotin anti-mouse IFNγ Antibody (clone XMG1.2, Biolegend, 505804) followed by Alexa Fluor 488-conjugated streptavidin (Thermofisher molecular probes, S-11223).

### Flow cytometry

Single cell suspension of splenocytes, lymph node, and peritoneal lavage were incubated with 10 μg anti-mouse unlabeled CD16/32 (2.4G2); stained with B220-APC or PeCy5, CD3-PE or PECy7, CD11b-FITC, or GL7-FITC (Biolegend), washed, examined on a BD Canto Flow analyzer, and data analyzed with FCS Express, v. 4.

### Immunohisto-, cytochemistry, and confocal microscopy

Ten μm OCT-embedded frozen sections of WT and *Spag6KO* lymph nodes were fixed in ice-cold acetone for 10 mins then left to air-dry. The sections were re-hydrated and blocked with 2% horse serum, then labelled with 10 μg/ml FITC-conjugated T- and B-Cell Activation Antigen (clone GL7, BD Biosciences 553666), PE-conjugated Rat Anti-Mouse CD21/35 (Clone 7G6, BD Biosciences, 552957), and Alexa Fluor 647 rat anti-mouse CD4 Antibody (clone GK1.5, Biolegend, 100424) for 45 mins. Sections were thoroughly washed, mounted with anti-fade mounting medium, Vectashield (Vector Laboratories), and cover-slipped. The sections were examined with Leica TCS-SP2 AOBS confocal laser scanning microscope fitted with an oil plan-apochromat ×40 objective. Three lasers were used: Argon (488 nm) for FITC, HeNe (543 nm) for PE, and HeNe (633 nm) for Alexa Flour 647 (shown as pseudo-color blue). Parameters were adjusted to scan at 1024 × 1024 pixel density and 8-bit pixel depth. Emissions were recorded in three separate channels, and digital images were captured and processed with Leica Confocal and LCS Lite software. The number, size and CD4 content of GCs in the LNs of WT and *Spag6*KO mice were counted and measured, and the average was presented + SD.

Leica Application Suite Advanced Fluorescence (LAS AF) was used to analyze the immunohistochemistry studies. The software measures area, length, number, position, density, and a range of more specialized parameters. First, a threshold was set for red, green, and blue intensity values that were used to distinguish objects of interest in the images. Initially, we set an arbitrary threshold that was later adjusted to the best threshold possible for the objects of interest. The same threshold was used strictly throughout the measurements to enable reliable comparisons. Two lymph nodes per mouse (4–5 *Spag*6KO or WT mice) were sectioned and used to quantify the number of GCs, size of GCs, and the number of GC CD4 cells. For GC measurement, the objects of interest were selected by drawing an enclosed circle and the system measures the selected areas as square micrometers. The number of CD4^+^ cells in the GC was also counted. The average GL-7-positive GC size, and CD4^+^ cells were determined by adding up GL-7-positive GC sizes and number of CD4 cells in follicles in all lymph nodes and then dividing the sum by the number of GCs examined.

For immuno-cyto-chemistry, WT or *Spag6*KO splenocytes were co-cultured with irradiated Balb/c splenocytes for 5 days, remaining live cells seeded into fresh IL-2 supplemented cRPMI for 3 days, and then allogeneic CTLs were cultured briefly with P815 target cells. Cells were transferred to pre-coated (ICAM-Fc, 1 μg/mL, R&D) Nunc chamber slides (Thermo Scientific), fixed, left to dry then blocked with 2% horse serum. The cells were labeled with Alexa Fluor 488 Phalloidin to visualize F-actin filaments at the synapse (thermofisher molecular probes, A12379) or mouse anti gamma-tubulin (clone GTU-88, Sigma-Aldrich T6557-.2 ML) for 45 mins then washed. Alexa Flour 488-conjugated Fab’2 fragment donkey anti-Rabbit IgG (H + L) (Jackson Immuno-Research 711-546-152) or anti Mouse IgG (H + L) (Jackson Immuno-Research 715-546-150) [with minimal cross reactivity to horse, mouse, rabbit, goat, human and rat proteins] secondary antibodies were then added together with rat anti-mouse CD8a-PE (southern Biotech, 1550-09 L) and NucRed 647 nuclear stain (Invitrogen, R37113). After 45 mins, the cells were thoroughly washed, mounted with anti-fade mounting medium, Vectashield (Vector Laboratories), and cover-slipped. The cells were examined with Zeiss Laser Scanning Microscope LSM 710 fitted with an oil plan-apochromat ×40, ×63, and ×100 objectives. Three lasers were used: Argon (488 nm) for FITC, HeNe (543 nm) for PE, and HeNe (633 nm) for NucRed 647 (shown as pseudo-color blue). Parameters were adjusted to scan at 1024 × 1024 pixel density and 8-bit pixel depth. Emissions were recorded in three separate channels, and digital images were captured. Z stacks running through the immunological synapse with 0.40 μm step-size were generated and re-structured into 3-dimensional snap shots and movies using Zeiss Zen 2012 (Blue Edition) software.

### Quantification of actin clearance and centrosome polarization

CD8 and P815 target cells were incubated in suspension for 5 min, plated onto glass multiwell slides, and incubated for a further 10 min (actin reorganization) or 20 min (centrosome polarization) at 37 °C. The cells were then fixed and stained by using antibodies against CD8, γ-tubulin, and F-actin. X-Y confocal sections and *en face* stack constructions through the synapse (supplementary movies 1 and 2 and still images (not shown)) were generated. Actin clearance was scored as “rings/cleared” when actin is totally cleared at the synapse leaving a contiguous ring; any residual actin disrupting this ring was scored “intermediate” and when a solid wall of actin was observed across the synapse this was scored as “un-cleared”. Centrosome docking was scored as “docked” when the centrosome was <1 μm from the synapse; “proximal” when on the synapse side of the nucleus within 1–3 μm and “distal” when >3 μm from the synapse. Data were calculated from 70–100 cell conjugates where CD8 cells were obtained from 4–5 *Spag*6KO or WT mice, and the experiment was repeated twice.

### Cytotoxic assay

CD8^+^ T cells were isolated from spleen using EasySep mouse PE selection Kit (Stemcell Technologies). CD8 T cells were cultured with platebound anti-CD3ε and anti-CD28 as described above for 2 days and then seeded into fresh media daily and used 6 days later. P815 (ATCC) target cells were grown in complete RPMI. Cytotox 96 Non-Radioactive Cytotoxicity Assay (Promega) was used to determine optimal P815 concentration (1.2 × 10^5^ cells/mL) and perform cytotoxicity assay.

### Centrosome staining

HEK293-T cells, either non transfected, or transfected with SPAG6/pcDNA3 plasmid and 48 hours post transfection cells were permeabilized, blocked, incubated overnight at 4 °C with a polyclonal anti-SPAG6 and monoclonal anti-γ-tubulin, washed, and incubated for 1 h at room temperature with Alexa 488-anti-mouse IgG (Jackson Immuno Research Laboratories) and Cy3-anti-rabbit IgG (Jackson ImmunoResearch Laboratories). Slides were washed and sealed using VectaMount with 4’, 6-diamidino-2-phenylindole (DAPI) (Vector Laboratories). Images were taken by confocal laser-scanning microscopy (Leica TCS-SP2 AOBS). To visualize the centrosomal association with SPAG6 in WT mice, T and B cells were isolated from splenocytes using Pan T Cell Isolation Kit II, mouse (Miltenyi Biotech, 130-095-130), and Pan B Cell Isolation Kit, mouse (Miltenyi Biotech, 130-095-813) respectively. The cells were fixed using reagent A of Fix and Perm Kit (Thermo Scientific, GAS003) then incubated overnight at 4 °C in permeabilization buffer B, and 10 μg/ml rabbit anti-peptide antibody generated against amino acid residues 438–452 (KVLPHDSKARRLFVT) in the C-terminus of murine SPAG6 that we have reported before^1^,^4^, and mouse anti γ-tubulin (clone GTU-88, Sigma-Aldrich T6557-.2 ML). After washing, the cells were incubated at room temperature for 1 hour with Alexa Flour 488-conjugated Fab’2 donkey anti Mouse IgG (H + L) (Jackson Immuno-Research 715-546-150) and Alexa flour 594 anti-Rabbit IgG (H + L) (Jackson Immuno-Research 711-586-152) in T cells, or Alexa flour 647 anti-Rabbit IgG (H + L) (Jackson Immuno-Research 711-606-152), shown as pseudo blue) in B cells. The cells were washed, cytospun, cover-slipped and examined with Zeiss Laser Scanning Microscope LSM 710 fitted with an oil plan-apochromat ×40, ×63, and ×100 objectives. Negative controls where only γ-tubulin primary antibody is used, followed by both secondary antibodies and using the same imaging parameters as for the non-transfected HEK cell images were included. SPAG6 signal in HEKs was also amplified using biotin-conjugated donkey anti-rabbit IgG (Jackson Immuno-Research, 711-065-152) followed by Streptavidin-Alexa Fluor 594 (Molecular Probes, S-11227) and compared with the non-amplified labeling.

### qPCR

Total RNA was isolated using TRIzol reagent (Invitrogen), reverse transcribed using RETROscript (Ambion, AM1710), and PCR performed to examine *Spag6* mRNA expression compared to *Gapdh and 18s* controls. The primer sets used in our previous study were applied^36^ and at least three replicates were analyzed. qPCR was run on an ABI 7500 Thermocycler (Applied Biosystems) using the SYBR-green system from Bio-Rad. The stability of 18s and GAPDH was compared using RefFinder^43^ which integrates the major computational algorithms geNorm^44^, Normfinder^45^, BestKeeper^46^, and the comparative Ct method^47^. Based on the RefFinder algorithm, the comprehensive Geomean ranking values of the tested reference genes recorded 1.189 and 1.414 for 18s and GAPDH respectively. Since lower ranking indicates more stable expression, 18s was selected for qPCR data normalization and presented with standard deviation between PCR replicates. *Spag6* normalized to GAPDH is shown in supplementary material.

The relative expression of *Spag6* to the internal controls GAPDH or 18s was calculated as 2^−deltaC^_T_ where delta Ct = C_T_ SPAG6 − C_T_ internal control^48^.

### Statistical analysis

Normal distribution of data sets was determined using Shapiro-Wilk normality test. The *p* values were calculated using unpaired two-tailed Student *t* tests in GraphPad Prism. Error bars represent the SEM between samples. A *p* value <0.05 is considered significant. In the cytotoxicity assays we used Mann-Whitney test to compare the difference on the averages and the mixed effects model to test the difference at the individual values. The mixed effects model^18^ was used to test the difference between wild and knockout mice, taking into account the variation between mice and within mice. Mouse type (WT or *Spag*6KO) and Effector/Target ratios (E:T ratios) were fitted as the fixed effects in the model, and the E:T ratios were also fitted as a random effect allowing the variation within each E:T ratio level.

## Additional Information

**How to cite this article**: Cooley, L. F. *et al.* Impaired immunological synapse in sperm associated antigen 6 (SPAG6) deficient mice. *Sci. Rep.* **6**, 25840; doi: 10.1038/srep25840 (2016).

## Supplementary Material

Supplementary Video 1

Supplementary Video 2

Supplementary Information
