# Supplementary material for: Impaired immunological synapse in sperm associated antigen 6 (SPAG6) deficient mice
Source: Sci Rep. 2016 May 12;6:25840. doi: 10.1038/srep25840 (PMC4864328; doi:10.1038/srep25840)
Supplement: Supplementary Information [file srep25840-s3.pdf]

Supplementary Information

Title: Impaired immunological synapse in sperm associated antigen 6 (SPAG6) deficient mice

Authors: Lauren Folgosa Cooley, Mohey Eldin El Shikh, Wei Li, Rebecca C. Keim, Zhengang Zhang, Jerome F Strauss III, Zhibing Zhang, and Daniel H. Conrad.

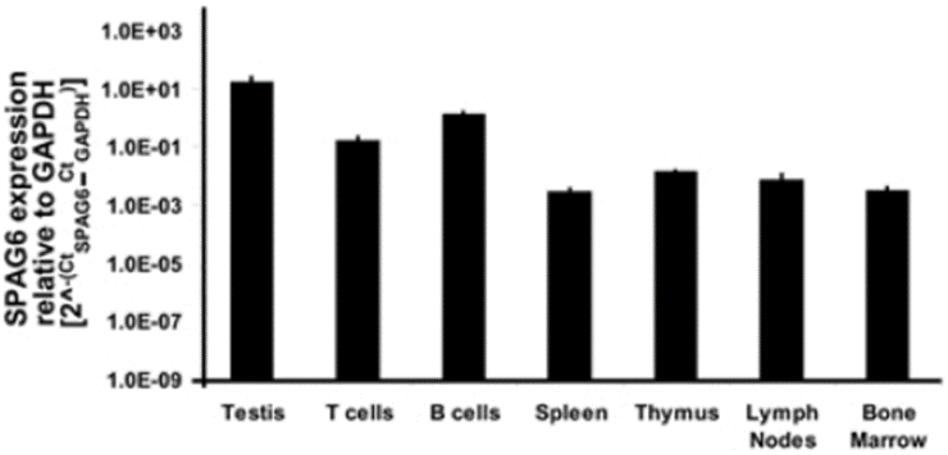

Supplementary figure 1: Quantitative Spag6 expression in different lymphoid tissues assessed by qRT-PCR and normalized relative to the GAPDH housekeeping gene.

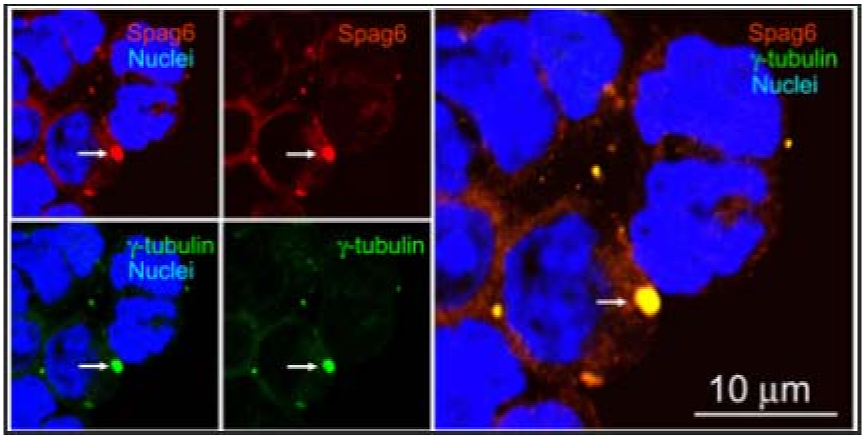

Supplementary figure 2: Non-transfected HEK293 cells were stained for DAPI (blue), SPAG6 (red) and γ-tubulin (green). The rabbit anti Spag6 signal was amplified using biotin-conjugated anti-aabbit IgG followed by Streptavidin-Alexa Fluor 594. Co-localization of endogenous SPAG6 and γ-tubulin is indicated by white arrows.

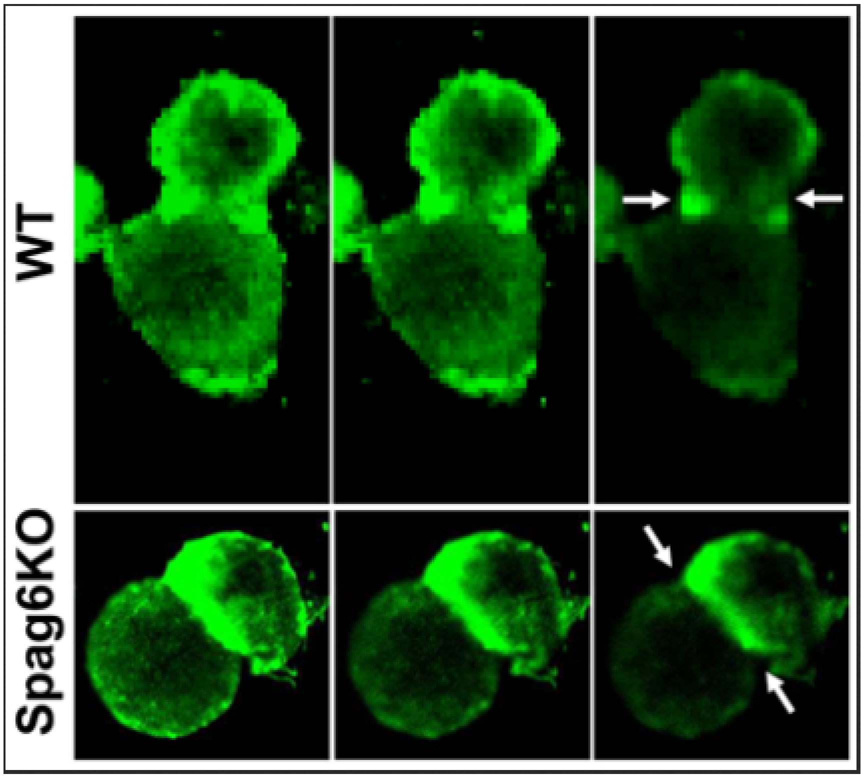

Supplementary figure 3: Serial still images taken from the supplementary movies of WT (movie S1) and Spag6 KO (movie S2), displaying variable amounts of actin labeling at different focal planes. Gradual display of the actin void at the immunological synapse of WT CD8 cells is shown from left to right. Defective actin clearance in the Spag6 KO CD8 cells resulted in persistence of strong actin signal at the immunological synapse at different focal planes.

Supplementary movie (S1). Movie constructed from serial confocal Z-stack showing α-actin (green) clearance at a normal immunological synapse between WT CD8+ CTL (red) and P815 target cell. Nuclei are shown blue.

Supplementary movie (S2). Movie constructed from serial confocal Z-stack showing defective α-actin (green) clearance at the immunological synapse between Spag6KO CD8+ CTL (red) and P815 target cell. Nuclei are shown blue.
